# Hepatitis B Virus Polymerase Blocks Pattern Recognition Receptor Signaling via Interaction with DDX3: Implications for Immune Evasion

**DOI:** 10.1371/journal.ppat.1000986

**Published:** 2010-07-15

**Authors:** Haifeng Wang, Wang-Shick Ryu

**Affiliations:** Department of Biochemistry, Yonsei University, Seoul, Korea; University of Southern California, United States of America

## Abstract

Viral infection leads to induction of pattern-recognition receptor signaling, which leads to interferon regulatory factor (IRF) activation and ultimately interferon (IFN) production. To establish infection, many viruses have strategies to evade the innate immunity. For the hepatitis B virus (HBV), which causes chronic infection in the liver, the evasion strategy remains uncertain. We now show that HBV polymerase (Pol) blocks IRF signaling, indicating that HBV Pol is the viral molecule that effectively counteracts host innate immune response. In particular, HBV Pol inhibits TANK-binding kinase 1 (TBK1)/IκB kinase-ε (IKKε), the effector kinases of IRF signaling. Intriguingly, HBV Pol inhibits TBK1/IKKε activity by disrupting the interaction between IKKε and DDX3 DEAD box RNA helicase, which was recently shown to augment TBK1/IKKε activity. This unexpected role of HBV Pol may explain how HBV evades innate immune response in the early phase of the infection. A therapeutic implication of this work is that a strategy to interfere with the HBV Pol-DDX3 interaction might lead to the resolution of life-long persistent infection.

## Author Summary

Viral infection is sensed by the host innate immune system, which acts to limit viral infection by inducing antiviral cytokines such as the interferons. To establish infection, many viruses have strategies to evade the innate immunity. For the hepatitis B virus (HBV), which causes chronic infection in the liver, the evasion strategy remains mysterious. An earlier study using the chimpanzee as a model suggested that the host innate immune system failed to detect HBV. As a result, it was dubbed “stealth virus”. In contrast, subsequent studies performed *in vitro* have suggested that HBV is, in fact, detected by the innate immune system but can effectively counteract this response. Whether HBV is detected by the innate immune system remains controversial; however, it is widely accepted that, regardless of detection, HBV effectively inhibits the host innate immune response early in infection through an unknown mechanism. The data presented here indicate that HBV Pol (polymerase or reverse transcriptase) blocks the innate immune response. This unexpected role of HBV Pol may explain why HBV appears to act as a “stealth virus” in the early phase of the infection.

## Introduction

Hepatitis B virus (HBV) is the prototypic member of the hepadnavirus family and a major cause of liver diseases. An estimated 400 million people are persistently infected with HBV worldwide. A significant subset of these HBV carriers progresses to severe liver disease, such as hepatocellular carcinoma, which may cause up to one million deaths per year. Interferon and nucleoside analogs such as lamivudine and adefovir are used to treat chronic hepatitis B patients but have limited utility due to the adverse effect and the emergence of drug-resistant variants, respectively [1]. Thus, there is a clear medical need for new therapeutic strategies.

Viral infection leads to the initiation of antiviral innate immune responses resulting in the expression of type I interferons (IFNs), IFNα and IFNβ, and pro-inflammatory cytokines [2]. Recently, the cellular mechanisms used to detect viruses and elicit production of IFNs and pro-inflammatory cytokines have been described in detail. It is now well-established that viruses, similar to bacteria and fungi, are initially recognized by host pattern-recognition receptors (PRRs) [2], [3]. Viral nucleic acids (both RNA and DNA) are the most important pathogen-associated molecular patterns (PAMPs) recognized by PRRs [3]. Two families of PRRs have been defined. The first is a subfamily of Toll-like receptors (TLRs) that include TLR3, TLR7, TLR8, and TLR9, which are mainly expressed in the endosomes of some cell types, especially plasmacytoid dendritic cells. Recognition by TLRs of viral PAMPs initiates TLR-mediated signaling pathways that culminate in the activation of transcription factors NFκB, IRF3, and IRF7. Specifically, TLRs recruit signaling adaptors, including TIR-domain-containing adaptor protein inducing IFNβ (TRIF). This activates TANK-binding kinase 1 (TBK1)/IκB kinase-ε (IKKε) to phosphorylate and activate the transcription factors IFN-regulatory factors (IRF) 3 and 7 [2], [4]. The second family of PRRs are comprised of the retinoic-acid inducible gene I (RIG-I)-like receptors (RLRs), including RIG-I and melanoma differentiation-associated gene 5 (MDA-5) [3]. Similar to TLRs, the recognition of viral nucleic acids by RLRs leads to a cascade of signaling events that result in activation of NF-κB, IRF3, and IRF7. Specifically, RLRs recruit the signaling adaptor protein IFNβ-promoter stimulator 1(IPS-1, also known as MAVS, VISA, or Cardif), activating the downstream TBK1/IKKε kinases, which then phosphorylate and activate IRF3 and IRF7 [2]. The capacity of both signaling pathways to restrict viral replication is consistent with their downstream convergence at the TBK1/IKKε kinases responsible for activation of IRF-3.

As stated above, viral infection leads to activation of cellular signaling such as IRF signaling, which culminates in IFN production. Infection by HBV appeared to be an exception. In an acute HBV-infected chimpanzee model, Chisari and colleagues have reported that HBV fails to induce transcription of any cellular genes that relate to the entry and expansion of the virus, implicating the lack of innate immune response upon HBV infection [5], [6]. By contrast to the earlier report, evidence was accumulating, which indicated that the innate immune system is, in fact, able to sense HBV infection. An early induction of innate immune response, as shown by the early development of natural killer cell and natural killer T cells response, was observed in two patients with acute viral infection [7]. More recently, by using a HepaRG cells, a permissive hepatocyte cell line for HBV infection, Zoulim and colleagues found that HBV infection elicits a strong innate antiviral response that leads to a significant reduction of HBV DNA synthesis [8]. Taken together, it is conceivable that one of viral proteins could impair innate immune response early in infection.

Two recent reports have shown that DDX3 DEAD box RNA helicase, which is known to be involved in diverse steps of RNA metabolism, could augment IRF signaling via its interaction with IKKε or TBK [9], [10]. In other words, DDX3 augments TBK/IKKε activity, which phosphorylates IRF3 and IRF7. Interestingly, our group has shown that DDX3 binds to HBV Pol (P protein) and inhibits viral reverse transcription [11]. Since DDX3 is essential for augmentation of IRF signaling, we postulated that HBV Pol impairs antiviral innate immune responses by inhibiting IRF signaling via its interaction with DDX3. Consistent with the notion, our results demonstrate that IRF signaling is significantly inhibited by HBV Pol, a finding that defines HBV Pol as a viral protein that counteracts antiviral pattern recognition receptor signaling.

## Results

### HBV Pol inhibits IFN promoter activity

**Figure 1:**
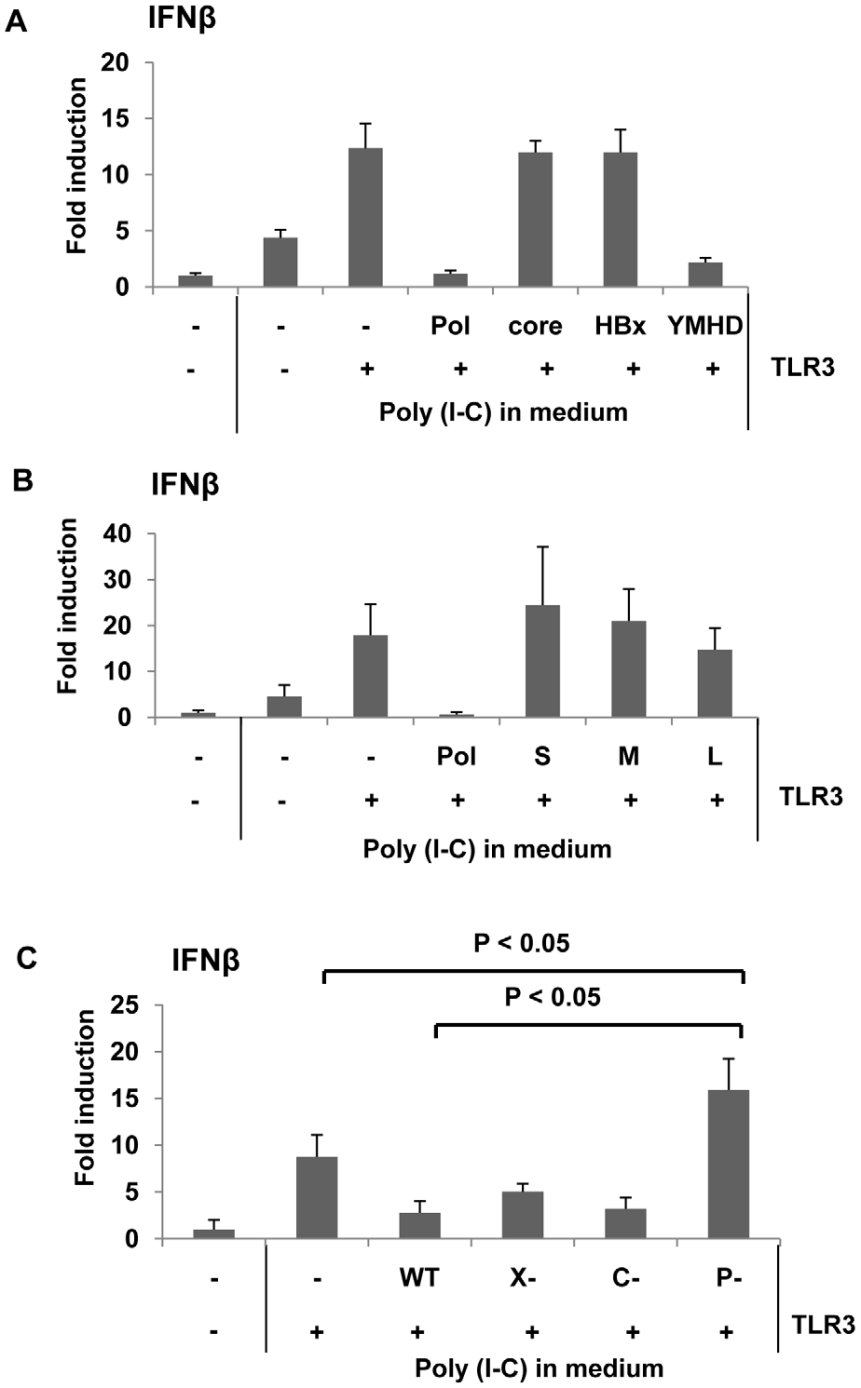
HBV Pol inhibits dsRNA-induced IFNβ promoter activation. (A) HepG2 cells in 6-well plate were transfected with HBV Pol, core, or HBx, and the YMHD mutant of HBV Pol expression constructs together with an IFNβ luciferase reporter and TLR3 expression constructs. Cells were stimulated with 25 µg/mL poly I∶C directly added to culture medium for 8 h. Data are expressed as the mean fold induction ± s.d. relative to mock-treated cells. The results are representative of at least four independent experiments. (B) An experiment was performed as shown in panel (A), with an exception of transfecting three viral surface antigen expression constructs: L-HBsAg (L), and M-HBsAg (M), and S-HBsAg (S). (C) HepG2 cells were transfected with empty vector, WT, X-null, C-null, and P-null HBV replicon together with the IFNβ reporter construct. Student's **t** test was used for statistical analysis, and a *P* value of <0.05 was considered statistically significant. Poly I∶C treatment was done as in panel (A). (See also Fig. S1).

To determine if HBV proteins could impair IRF signaling and counteract host innate immune responses to HBV infection, we tested the ability of HBV proteins to inhibit interferon (IFN) β promoter activity. Synthetic dsRNA mimic polyinosine-polycytidylic acid (poly I∶C) is recognized by TLR3 in endosomes when added to medium [12]. To induce IRF signaling, human hepatoma HepG2 cells, which support viral genome replication, were transfected with one of three viral protein expression constructs — core, polymerase (Pol), or HBx —, a TLR3 expression construct , and a reporter construct expressing luciferase under control of the IFNβ promoter (Fig. 1A). Cells were complemented with the TLR3 expression construct because HepG2 cells are deficient in TLR3 expression [13]. To ensure that the physiological levels of viral proteins were attained, the amount of the three viral protein expression constructs for transfection were determined by cotransfection with the corresponding gene-null HBV replicon: for HBV Pol (see Fig. S2), for core protein (data not shown), and for HBx [14]. Two days following transfection, cells were treated with poly I∶C. Eight hours post-poly I∶C treatment, cells were harvested and luciferase activity was measured. As anticipated, the luciferase activity was significantly induced when cells were complemented with TLR3, compared to that induced by poly I∶C only (Fig. 1A). The data indicated that HepG2 cells were able to recognize extracellular dsRNA and induce IFN production when complemented with TLR3. More importantly, the data showed that HBV Pol, but not other viral proteins including HBx, significantly suppressed IFN promoter activity (Fig. 1A). The inhibition of IFN promoter activity by HBV Pol was somewhat unexpected, since current literature suggests that almost all of its known function is confined to the functions – encapsidation and viral reverse transcription – occurring inside of nucleocapsids [15]. Further, reverse transcriptase activity of HBV Pol appeared not to be involved in the inhibition of IFN promoter activity, as the YMHD mutant of HBV Pol, reverse transcriptase activity deficient mutant, remained to inhibit IFN promoter activity (Fig. 1A). In addition, we found that three viral envelope glycoproteins– L-HBsAg, and M-HBsAg, and S-HBsAg– had no impact on IFN promoter activity (Fig. 1B), indicating that HBV Pol is the only viral protein that has an inhibitory effect on IFNβ production.

To substantiate the above results in the context of viral life cycle, the impact of viral proteins on IFNβ production was examined by using a viral replicon, which could lead to viral genome replication when transfected [14]. Three mutants were made in which one viral gene was inactivated per construct: (i) P (Pol)-null, (ii) C (core)-null, and (iii) X-null (Fig. S1). An increase in IFNβ production by one of the HBV mutant constructs would point out that particular gene in the inhibition of IFN production. Luciferase activity was monitored following transfection as described above. The data revealed that only cells transfected with the HBV P-null replicon construct induced a higher level of IFN production, whereas the other three replicons, including the wild-type, induced modest level of IFN production (Fig. 1C). These results suggested that HBV Pol derived from the HBV replicon – wild-type or X-null, or C-null replicon – decreased IFN production, implicating the physiological relevance of the findings in the viral life cycle. Intriguingly, IFN promoter activity induced by HBV P-null replicon was significantly higher than what was achieved by poly I∶C (Fig. 1C). The implication is that some yet-to-be known viral PAMPs derived from HBV P-null replicon could contribute to the augmented IFN promoter activity (see Discussion).

### HBV Pol inhibits IFNβ promoter triggered by RIG-I/MDA5 as well as TLR3 receptor signaling

**Figure 2:**
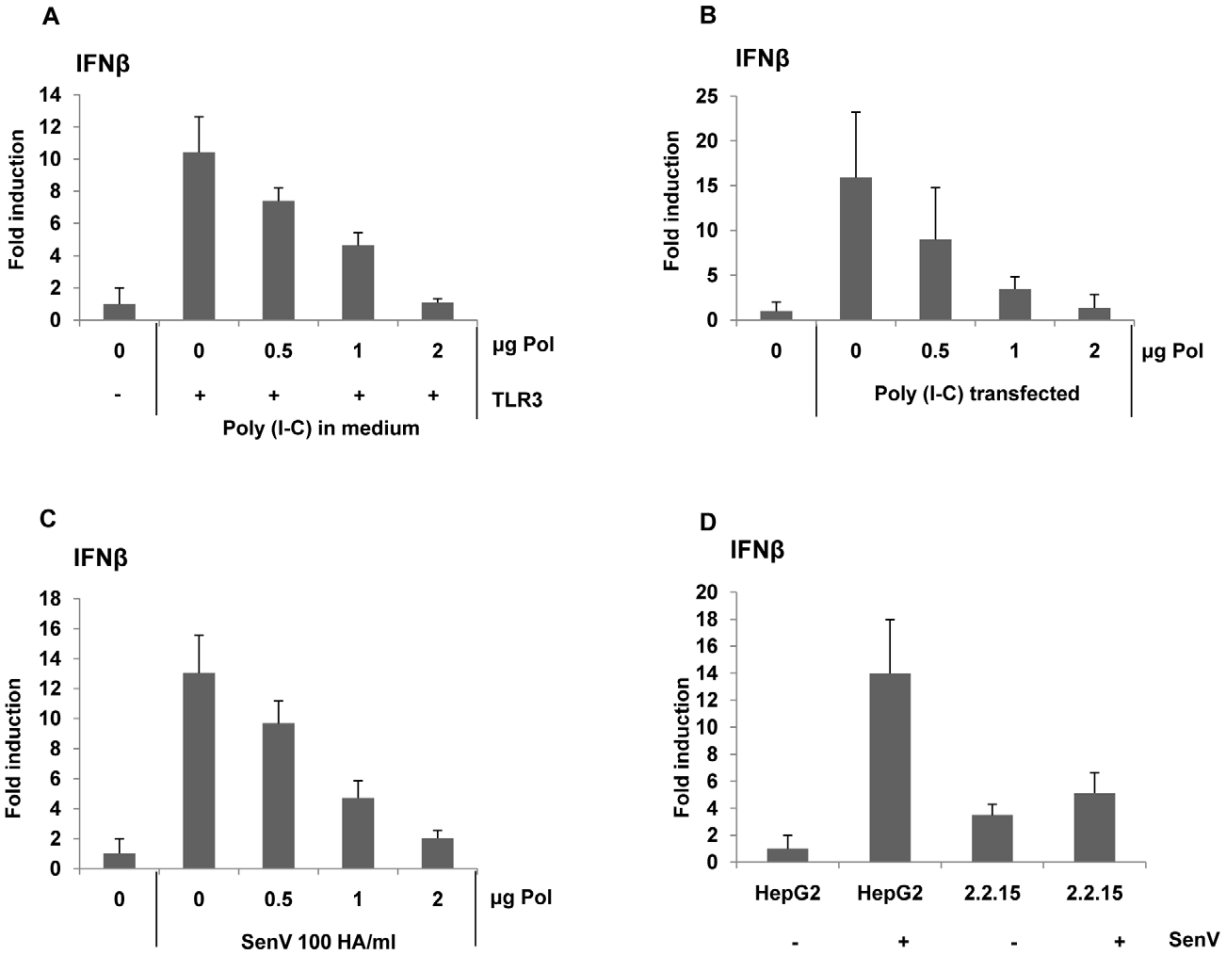
HBV Pol inhibits both dsRNA- and Sendai virus-induced IFNβ promoter activation. HepG2 cells were transfected with increasing doses of the HBV Pol expression construct, along with the IFNβ luciferase reporter construct and TLR3 expression construct. Cells were stimulated with 25 µg/mL poly I∶C directly added to the medium for 8 h (A), with 25 µg/mL poly I∶C transfected with lipofectin for 8 h (B), or with Sendai virus (SenV) 100 HA/mL for 6 h (C). Data are expressed as the mean fold induction ± s.d. relative to control levels. The results are representative of three independent experiments, each performed in triplicate. (D) Cells (HepG2 or HepG2.2.15) were transfected with an IFNβ luciferase reporter and then, infected with Sendai virus (SenV) 100 HA/mL for 6 h before harvest.

To examine whether HBV Pol could suppress diverse PAMP-mediated signaling, poly I∶C was given to cells by two distinct routes: (i) addition to medium, and (ii) transfection via lipofectin. The poly I∶C is recognized by TLR3 in endosomes by adding it to the cell medium, whereas it is recognized by MDA5 when it is delivered to the cytoplasm by transfection via lipofectin [16], [17]. Cells were transfected with incremental dose of the HBV Pol and TLR3 expression constructs along with the reporter construct. Following treatment with poly I∶C in the medium, the reporter assay result showed a dose-related decrease of IFN promoter activity by HBV Pol expression, corroborating the above conclusions (Fig. 2A). Likewise, when poly I∶C was given by lipofectin transfection, IFN promoter activity was similarly decreased by HBV Pol (Fig. 2B).

Next, innate immune response was triggered by Sendai virus (SenV), a potent stimulus of the RIG-I pathway [17], [18]. Cells were transfected with the HBV Pol expression construct and the IFN-luciferase construct. Two days after transfection, cells were treated with 100 HA U/mL of SenV for 6 h before harvest. The data indicated that HBV Pol diminished IFN promoter activity in a dose-related manner (Fig. 2C). Overall, the data presented suggest that HBV Pol suppresses RIG-I mediated IFN production as well as TLR3-mediated IFN production.

To substantiate the above findings, we wanted to eliminate a possibility that the reduction of the IFN promoter activities by HBV Pol is ascribed to the over-expression of HBV Pol. To assess whether the HBV Pol we expressed ranges the physiological level, we sought to show that the amount of viral DNAs synthesized via complementation by the HBV Pol in the HBV P-null replicon transfected cells is comparable to that of WT HBV replicon transfected cells. To this end, HepG2 cells were transfected with the same four increasing amount of the HBV Pol expression construct as above to complement the P-null construct (i.e., P-) for the viral genome replication. Viral DNAs extracted from cytoplasmic capsids were measured by Southern blot analysis (Fig. S2). The data showed that the amount of viral DNA synthesized by complementation was less than that of WT HBV replicon (Fig. S2, lane 1 versus lanes 3 to 5), suggesting that the HBV Pol expressed in the Fig. 2A to Fig. 2C was not exceeding physiological level. In addition, by using HepG2.2.15 cell line that stably expresses viral proteins and support HBV replication [19], we consistently found that IFN promoter activity induced upon SenV (Sendai virus) infection was pronouncedly diminished in HepG2.2.15 cells (Fig. 2D), validating the impact of HBV Pol in a more physiological setting.

### HBV Pol abrogates nuclear translocation and phosphorylation of IRF3

**Figure 3:**
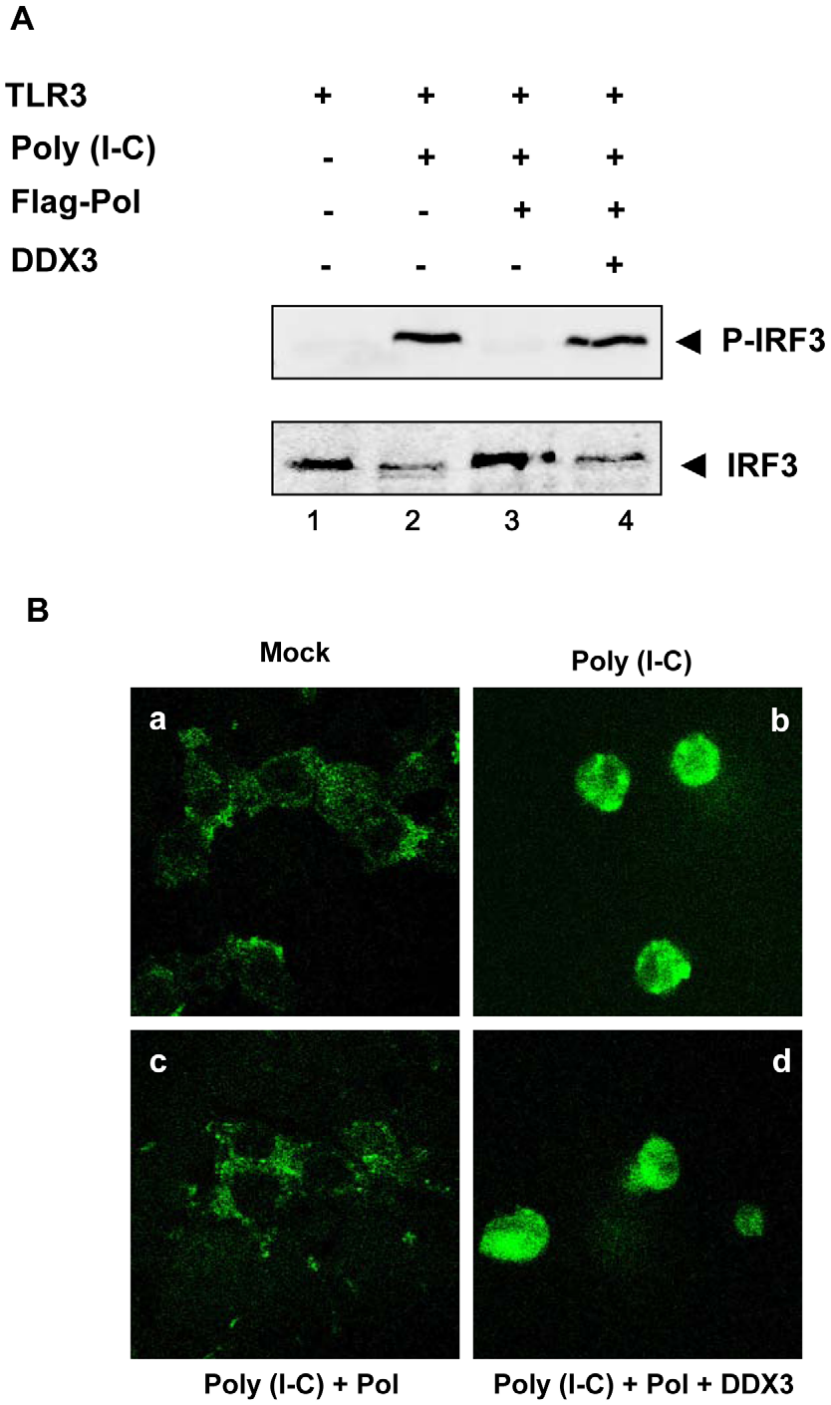
HBV Pol inhibits both phosphorylation and nuclear translocation of IRF3. (A) HEK293 cells were transfected with TLR3, HBV Pol, and DDX3 expression construct as indicated. Two days posttransfection, cells were stimulated with poly I∶C for 8 h. Western blot analysis was performed as described in Materials and Methods. (B) HEK293 cells were transfected with GFP-IRF3, TLR3, HBV Pol, and DDX3 expression constructs, as labeled either above or below each panel by a, b, c, and d. Two days posttransfection, cells were treated with poly I∶C for 8 h. Cells were examined by confocal microscopy.

Phosphorylation and nuclear translocation of IRF3 represent hallmarks of antiviral innate immunity. To examine whether HBV Pol inhibits the phosphorylation of IRF3, cells were transfected with TLR3 and HBV Pol construct as indicated. Eight hours before harvest, cells were then treated with poly I∶C. Endogenous IRF3 and its phosphorylated counterparts were detected by Western blot analysis with anti-IRF3 and anti-phosphorylated IRF3 (Ser396) antibodies, respectively (Fig. 3A). As anticipated, Western blot analysis indicated that the higher molecular weight bands of endogenous IRF3, which represent phosphorylated IRF3, appeared when cells were treated with poly I∶C (Fig. 3A, lane 2). However, the phosphorylated IRF3 was undetectable following HBV Pol expression (Fig. 3A, lane 3), suggesting that HBV Pol inhibits phosphorylation of IRF3. (see below for the explanation for lane 4).

Nuclear translocation of IRF3 was examined by fluorescence microscopy. Cells were transfected with the IRF3-GFP fusion protein construct to monitor IRF3 nuclear localization (Fig. 3B). Cells were also transfected with HBV Pol and TLR3 constructs and then treated with poly I∶C. As previously demonstrated [20], IRF3-GFP predominantly found in the cytoplasm was induced to undergo nuclear localization only when cells were treated with poly I∶C (Fig. 3B, panel a versus panel b). By contrast, IRF3-GFP remained localized in cytoplasm when cells were transfected by HBV Pol and treated with poly I∶C (Fig. 3B, panel c), suggesting that HBV Pol prevents the nuclear translocation of IRF3-GFP. Overall, these data are consistent with the conclusion that HBV Pol inhibits the IRF signaling. (see below for the explanation for panel d).

### HBV Pol suppressed both TRIF and RIG-I triggered IRF signaling

**Figure 4:**
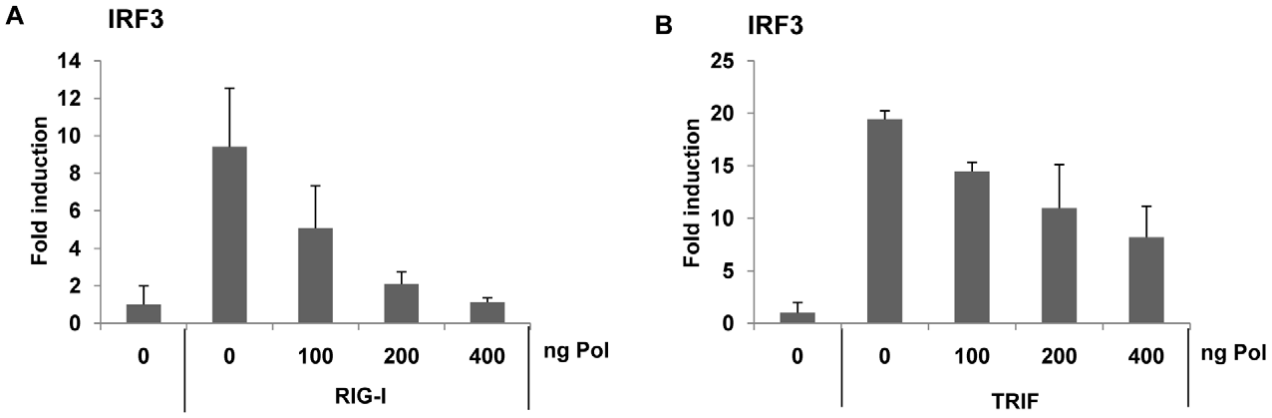
HBV Pol inhibits both RIG-I- and TRIF-induced IRF3 activation. HepG2 cells were cotransfected with increasing doses of the HBV Pol expression construct to determine the effect of HBV Pol on IRF3 signaling. To monitor IRF3 activation, cells were transfected with IRF3-GAL4 expression plasmid together with the GAL4-dependent pFR luciferase reporter construct. Note that 5-fold less amount of HBV Pol construct was used, because 24-well plate, instead of 6-well, was used. To induce IRF3 signaling, RIG-I (A) or TRIF (B) expression constructs were transfected. Data are expressed as the mean fold induction ± s .d. relative to control levels. The results are representative of at least two independent experiments, each performed in triplicate.

To further elucidate the specific mechanism by which viral HBV Pol interferes with IRF activation, the impact of HBV Pol on the signaling pathway leading to IRF activation was investigated. To trigger RIG-I mediated IRF3 signaling, RIG-I was over-expressed. On the other hand, TRIF is an adaptor for the TLR3 receptor and mimics TLR3 signaling when over-expressed. To trigger TLR-mediated IRF3-signaling, TRIF over-expression was employed. Cells were transfected with the IRF3 reporter construct and an incremental dose of HBV Pol expression construct, along with either RIG-I or TRIF (Fig. 4A and Fig. 4B). The data indicated that the luciferase activity was decreased by HBV Pol in a dose-dependent manner (Fig. 4A and Fig. 4B). Thus, we concluded that HBV Pol inhibited both TLR-mediated and RIG-I-mediated IRF3 signaling.

### HBV Pol suppressed TBK1/IKKε triggered IRF 3/7 signaling

**Figure 5:**
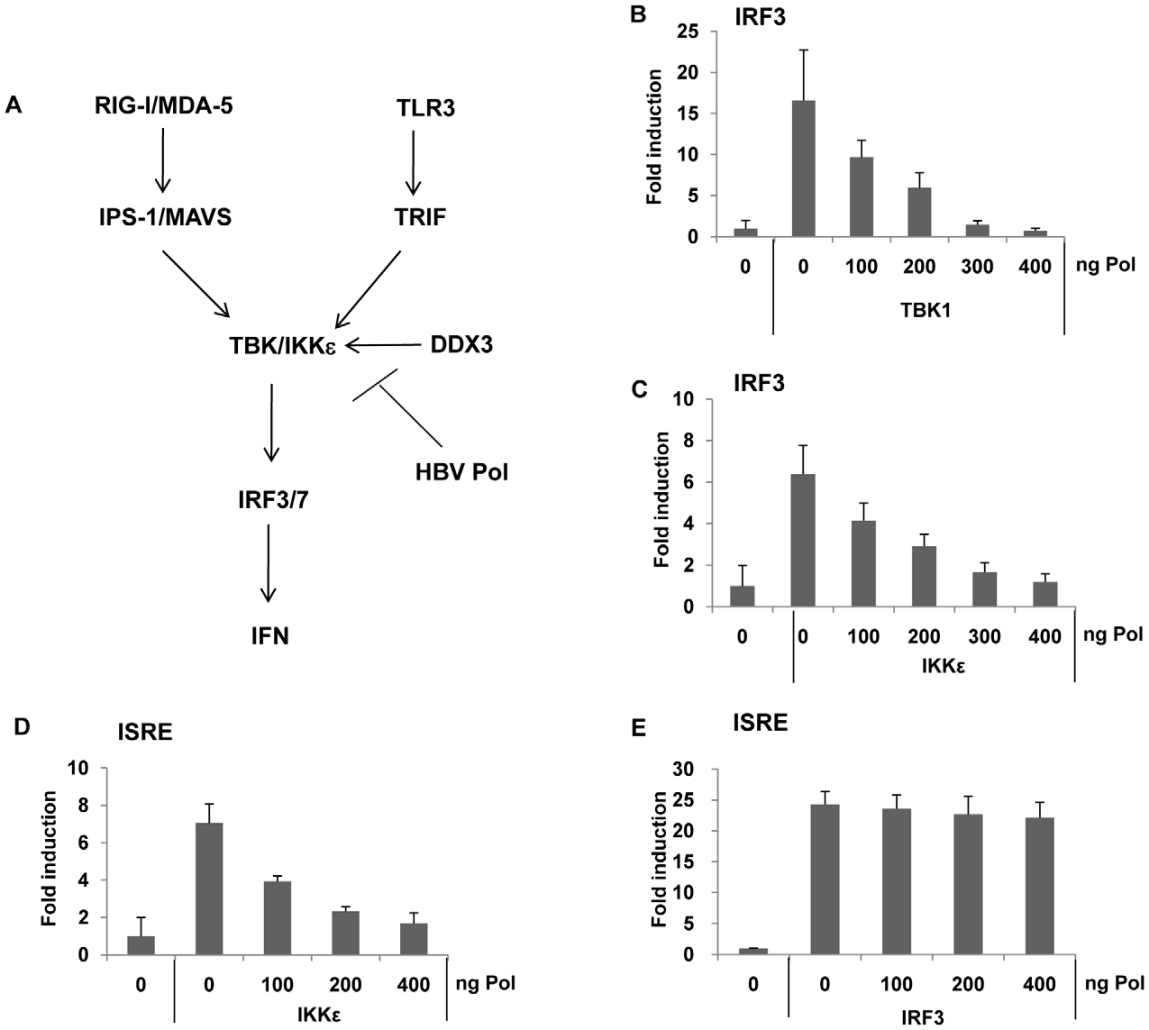
HBV Pol blocks IRF activation by inhibiting TBK1/IKKε activity. (A) Schematic of IRF signaling pathway leading to IFN production. TBK1 serves as a central effector kinase, but TBK1 function is also shared by the highly related IKKε kinase in some cells. DDX3 is described in an experiment shown in Fig. 6 (*see text*). (B and C) HepG2 cells were cotransfected with an incremental amount of HBV Pol expression construct to determine the effect of HBV Pol on IRF3 signaling. To induce IRF3 signaling, or TBK1 (B) or IKKε (C) expression constructs were transfected. IRF3 activation was monitored as shown in Fig. 4. (D and E) Cells are transfected as in (B and C), respectively, except that ISRE-luciferase reporter construct was transfected, instead of the IRF reporter construct.

The TBK1/IKKε complex represents the effecter protein kinase of IRF signaling, phosphorylating IRF3/7, when activated by appropriate recruitment of IPS/MAVS or TRIF (Fig. 5A). The results shown above indicated that both TLR3- or RIG-I-mediated IRF signaling was inhibited by HBV Pol, and the phosphorylation of IRF3/7 was blocked by HBV Pol. Thus, it is conceivable that HBV Pol inhibits TBK1/IKKε activity. To gain further insight into the mechanism by which HBV Pol interferes with TLR3-mediated and RIG-I-mediated IRF signaling, we determined if TBK1 or IKKε triggered IRF signaling could also be blocked by HBV Pol. Cells were transfected with either TBK1 or IKKε, and IRF3 reporter construct along with incremental doses of the HBV Pol construct. Results indicated that IRF3 signaling was triggered either by TBK1 or IKKε over-expression and was blocked by HBV Pol in a dose-dependent manner (Fig. 5B and 5C). Essentially identical data were obtained with an IRF7 reporter assay performed in parallel (data not shown). Thus, the data suggested that HBV Pol exerts its inhibitory effect on TBK1/IKKε or another downstream point in this pathway. An ISRE reporter assay was then carried out, which indicated that HBV Pol inhibited IKKε-triggered ISRE activation, but not IRF3-triggered ISRE activation (Fig. 5D and 5E). These results pointed to TBK1/IKKε as the molecular target of HBV Pol for the inhibition of IRF signaling.

### HBV Pol inhibits TBK1/IKKε activity by disrupting IKKε-DDX3 interaction

**Figure 6:**
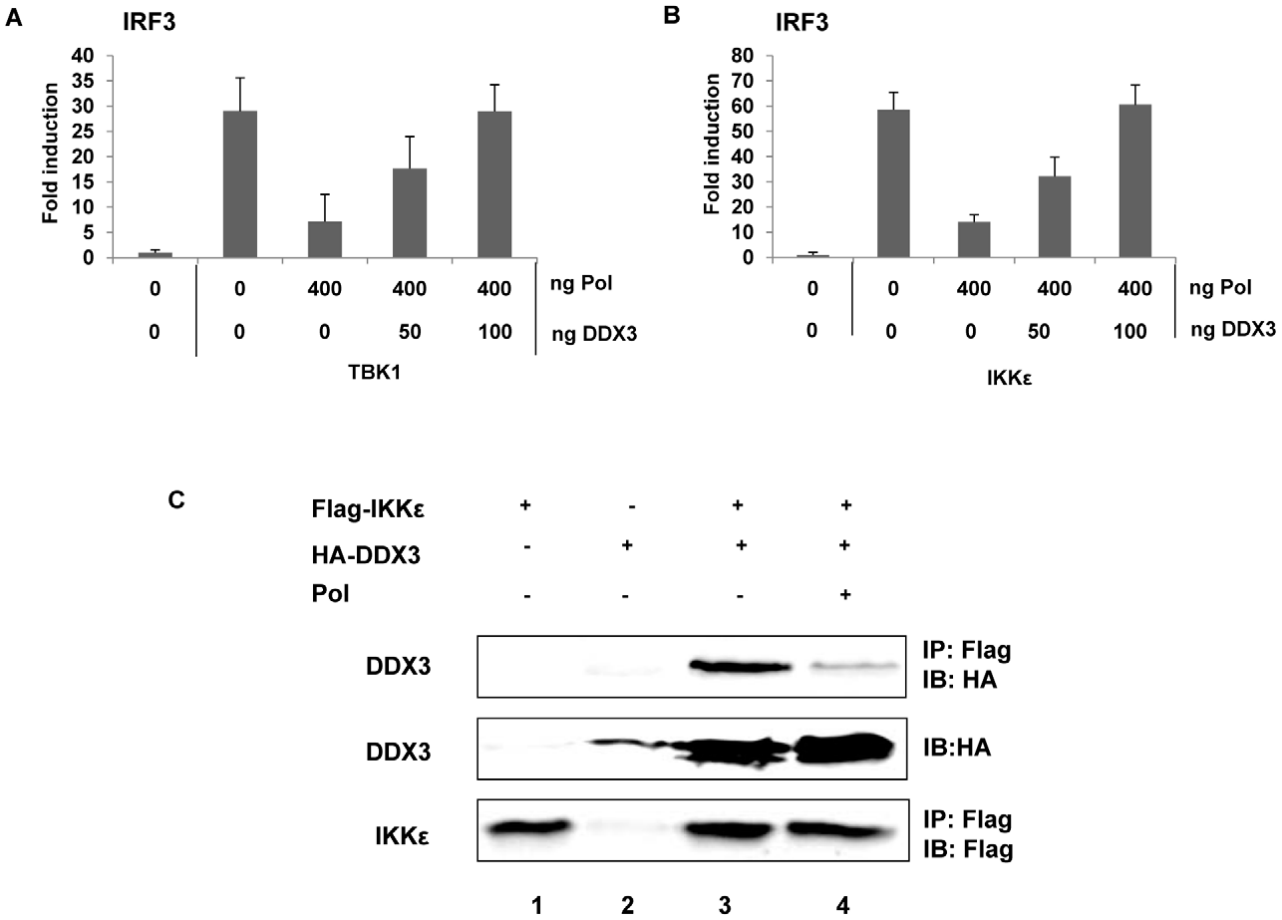
HBV Pol inhibits TBK1/IKKε via an interaction with DDX3. (A and B) DDX3 rescued IRF signaling, which was inhibited by HBV Pol. HepG2 cells were transfected with indicated amounts of HBV Pol and DDX3 expression constructs. To induce IRF3 signaling, TBK1 (A) or IKKε (B) expression constructs (100 ng) were transfected. IRF3 activation was monitored as shown in Fig. 4. Data are expressed as the mean fold induction ± s.d. relative to control levels. The results are representative of at least two independent experiments each performed in triplicate. (C) HBV Pol disrupted the interaction between DDX3 and IKKε. HEK 293 cells were transfected with Flag-IKKε, HA-DDX3, and HBV Pol expression constructs. Cell lysates were analyzed by IP with the indicated antibodies to assess the interaction between DDX3 and IKKε. For the detection of the Flag-tagged IKKε, mouse anti-FLAG M2 antibody (Sigma, 1∶5,000) was used; anti-HA antibody (Amersham) was used to detect DDX3. The results are representative of three independent experiments.

Next, we examined whether HBV Pol inhibits TBK1/IKKε activity directly or indirectly. Recently, two independent groups demonstrated that DDX3 enhanced TBK1/IKKε activity via its interaction with TBK1 or IKKε [9], [10]. Additionally, we have shown that DDX3 binds to HBV Pol [11]. Thus, we hypothesized that HBV Pol would inhibit TBK1/IKKε activity via interaction with DDX3. One prediction of the hypothesis is that over-expression of DDX3 would restore IRF signaling that has been inhibited by HBV Pol. Previous studies were limited to HEK293 and RAW264.7 macrophages; therefore, we confirmed the importance of DDX3 for the activation of IRF signaling in HepG2 cells following downregulation of DDX3 (Fig. S3A and Fig. S3B). To determine if DDX3 could restore IRF3 signaling suppressed by HBV Pol, cells were transfected by either TBK1 or IKKε constructs along with a maximal level of HBV Pol to obtain the highest level of IRF signaling inhibition. Cells were also cotransfected with increasing doses of the DDX3 construct to determine if DDX3 could restore the diminished IRF signaling. The luciferase data revealed that the ectopic expression of DDX3 rescued the IRF signaling in a dose-dependent manner (Fig. 6A and 6B), indicating that DDX3 antagonizes the inhibitory effect of HBV Pol on IRF signaling. Consistently, ectopic expression of DDX3 also rescued the phosphorylation and nuclear translocation of IRF3 (Fig. 3A, lane 4; Fig. 3B, panel d).

Given the functional interaction between HBV Pol and DDX3 [11], it is possible that HBV Pol disrupts the IKKε-DDX3 interaction. To assess this possibility, a co-immunoprecipitation (co-IP) following transfection of Flag-IKKε, HA-DDX3, and HBV Pol expression constructs was performed (Fig. 6C). Similar to previous reports [9], an interaction between IKKε and DDX3 was observed (Fig. 6C, lane 3). Importantly, when the HBV Pol construct was cotransfected, the IKKε-DDX3 interaction was significantly diminished, consistent with an interpretation that HBV Pol disrupts the IKKε-DDX3 interaction (Fig. 6C, lane 4). It was also noted that DDX3 level was enhanced and a higher molecular weight form of DDX3 was evident when IKKε was ectopically expressed, which might represent a phosphorylated form (Fig. 6C, lanes 3 and 4); intriguingly, the similar changes of DDX3 were also observed in the previous report [9]. It is conceivable that the phosphorylation of DDX3 by IKKε stabilizes DDX3. Based on the antagonistic activity of DDX3 on the inhibitory effect of HBV Pol on IRF signaling and the disruption of IKKε-DDX3 interaction by HBV Pol, we concluded that HBV Pol suppresses IRF signaling by disrupting the IKKε-DDX3 interaction.

## Discussion

HBV, that has been dubbed a “stealth virus”, efficiently evades antiviral innate immune responses early in infection [6]. However, the underlying immune evasion mechanism remained enigmatic. The data presented here revealed that HBV evades host innate immune response via the inhibition of pattern recognition receptor signaling by one of the viral proteins. Results presented here clearly show that HBV Pol is the viral protein that blocks the TLR3-and RIG-I-induced pattern recognition receptor signaling in a physiologically relevant setting (Fig. 2). Further, evidence suggests that HBV Pol suppresses IRF signaling by inhibiting TBK1/IKKε activity, the effector protein kinase of IRF3/7 signaling (Fig. 3). Importantly, we also demonstrated that HBV Pol inhibits TBK1/IKKε activity by disrupting the interaction between IKKε and DDX3 (Fig. 6C). Overall, besides its inherent catalytic role in viral reverse transcription, our results here confer a novel immune-regulatory role to HBV Pol.

Recent evidence obtained in cultured cells showed that HBV is capable of inducing innate host response [8], [21]. However, it remained to be learned how HBV abrogates innate immune response. The data presented here indicate that HBV Pol blocks both TLR-mediated and RIG-I-mediated IRF3 signaling (Fig. 1 and Fig. 2). Importantly, two experiments presented here supported the physiological relevance of the impact of HBV Pol on innate immune response. First, the data in Fig. 1C showed that the inhibition of IRF signaling by HBV Pol is physiologically relevant, since the inhibitory effect by HBV Pol was observed under the condition, where the physiological level of viral proteins was attained by using either wild-type or mutant HBV replicon in HepG2 cells (Fig. 1C). This finding is further strengthened by the evidence that IFN production was only marginally induced upon SenV infection in HepG2.2.15 cell line compared to a parental HepG2 cells (Fig. 2D).

Further, the data shown in Fig. 1C is significant in a few respects. It was not unexpected that the extent of inhibition attained by the X-null and C-null construct was comparable to that by the wild-type replicon (Fig. 1C), since similar level of HBV Pol would be expressed in transient transfection setting, regardless of whether viral genome replication is induced. Secondly, the data clearly ruled out a possibility that either X protein (i.e., HBx) or core protein is related to the inhibition, since IFNβ production in either X-null or C-null transfected cells was comparable to that of WT transfected cells (Fig. 1C). Thirdly, the cells transfected by the P-null replicon mounted significantly and reproducibly higher IFN production than that by mock DNA, suggesting that a viral molecule derived from the P-null replicon caused augmentation of IFNβ production, in addition to what has already been induced by poly(I∶C) (Fig. 1C). It is tempting to speculate that the viral molecule, perhaps viral RNAs, may represent viral PAMPs that caused enhanced IFNβ production.

Although the liver is an important site for chronic viral infection, little is known about how innate immune response is initiated in hepatocytes [13], [22]. We chosen HepG2 cell for the most experiments, a hepatoma cell line, that supports HBV genome replication in a manner depending on HBx expression [14]. Since TLR3 expression is deficient in HepG2 cells, IFNβ reporter assays were carried out following complementation of TLR3; note that HepG2 cell is proficient in RIG-I mediated signaling, which senses SenV [13]. On the other hand, unlike to most other viruses, the PAMP signature of HBV, which are sensed by pattern recognition receptors such as TLRs or RIG-I, has not been defined [2]. Consequently, our analysis was limited to use of poly I∶C or SenV to trigger pattern recognition receptor signaling. However, it should be noted that the utilization of heterologous inducers such as poly I∶C or SenV in this work does not invalidate our conclusions, since the HBV Pol inhibits TBK/IKKε kinase, which lies downstream of PAMPs in IRF signaling (Fig. 5A). Nonetheless, natural viral infection, rather than transfection, involving susceptible human hepatocytes, merit further investigation, as recently demonstrated in HCV infection [23].

In terms of the impact of HBV Pol on IFN signaling, three observations are worthy of mentioning. First, our preliminary data indicated that in addition to its inhibitory effect on IRF signaling, HBV Pol inhibits, to a lesser extent, NF-kB signaling, which also contributes to IFNβ production (data not shown). If it holds, it would suggest an intriguingly possibility in that HBV Pol blocks IFNβ production by interfering two distinct signaling pathways, leading to IFNβ production. Work is in progress to obtain the mechanistic details. Second, relevantly, abundant detection of HBV Pol in nonencapsidated state has implicated that HBV Pol could contributes to viral pathogenesis or immune evasion [24], [25]. Lastly, in fact, HBV Pol has been previously identified as one of viral proteins that confer the resistance to IFN treatment [26], [27]. It should be noted, however, that we found its inhibitory role in IFN induction (i.e., IRF signaling), whereas the published work found its inhibitory effect in IFN action (i.e., JAK/Stat signaling).

DEAD-box RNA helicases constitute a large family of proteins that comprises at least 38 members in human genome [28]. DEAD-box RNA helicases exhibit multiple roles in diverse aspects of RNA metabolism such as transcription, pre-mRNA splicing, RNA export, translation, and RNA decay [29]. Not surprisingly, DDX3 has also been implicated in multiple distinct cellular processes as well. First, DDX3 has been reported to act as a transcriptional factor in the nucleus [30]. Secondly, DDX3 was shown to bind to eIF4E, a translation initiation factor with cap-binding properties, effectively suppressing translation [31]. Finally, DDX3 has been implicated in various viral life cycles. For instance, DDX3 was shown to be essential for nuclear export of human immunodeficiency virus-1 (HIV-1) RNA through the Rev/RRE pathway [32]. In addition, DDX3 supports viral replication of hepatitis C virus (HCV) genome via its interaction with the HCV core protein [33], [34]. In contrast, DDX3 was shown to inhibit HBV genome replication via its interaction with HBV Pol [11]. Besides its roles in RNA metabolism, a novel function relevant to innate immunity was recently reported by two groups [9], [10]. Although the augmentation of IRF signaling by DDX3 was found independently by two groups, discrepancies have been noted regarding the specific mechanism for DDX3-mediated IRF3 activation. Specifically, Bowie and colleagues [9] demonstrated that the IKKε-DDX3 interaction is significantly enhanced upon SenV infection with concomitant IRF3 phosphorylation, indicating that DDX3 stimulates the protein kinase activity of IKKε. In contrast, Decker and colleagues [10] concluded that DDX3 acts as a transcription factor of the IFNβ promoter, which is in agreement with the transcriptional role of DDX3 reported by another study [30], [35]. Interestingly, we have demonstrated that HBV Pol disrupts the IKKε-DDX3 interaction (Fig. 6C), which is consistent with the conclusions of Bowie and colleagues (7). However, in-depth analyses are needed to clarify this mechanistic issue.

Throughout evolution, viruses have developed strategies to evade host immune response [36]. Bowie and colleagues [9] revealed that the vaccinia virus K7 protein interferes with IRF signaling by inhibiting TBK1/IKKε activity via a mechanism involving its interaction with DDX3. Here, we demonstrated that HBV Pol impairs IRF signaling by inhibiting TBK1/IKKε activity via the HBV Pol-DDX3 interaction. Although diverse viral proteins including structural proteins and nonstructural proteins have evolved to evade immune response [36], it is intriguing that viral polymerase, besides its inherent catalytic contribution, has evolved to interfere innate immune response. More importantly, it is interesting that vaccinia virus and HBV, two unrelated viruses, acquired the ability to evade the immune response by subverting DDX3 during evolution.

Administration of TLR ligands was shown to inhibit HBV replication in a transgenic mouse model, implying that pattern recognition receptor signaling could be exploited for the treatment of chronic HBV infections [37]. Subsequent studies corroborated the above findings either by transfection studies using hepatoma cell lines [38] or by using nonparenchymal liver cells from mice [39]. Along this line, we speculate that disruption of the HBV Pol-DDX3 interaction by therapeutic intervention could invoke sustained antiviral immune responses leading to resolution of chronic viral infections. In this regard, structural elucidation of the HBV Pol-DDX3 interaction merits further investigation.

This work was presented at the International Meeting on Molecular Biology of Hepatitis B Viruses, which was held in Tours, France from August 30 to September 3 of 2009 [40] and the result similar to ours will be reported by others [41].

## Materials and Methods

### Cell culture, transfection

HepG2, HepG2.2.15, and HEK293 cells were grown in Dulbecco's Modified Eagle's medium supplemented with 10% fetal bovine serum (GIBCO-BRL) and 10 µg/mL gentamycin at 37°C in 5% CO_2_ and were passaged every third day. Cells were transfected using polyethylenimine (25 kDa, Aldrich, St. Louis, MO) as described [42]. The amount of plasmid DNA transfected (12 µg per 60-mm plate and 30 µg per 100-mm plate) was kept constant by inclusion of vector DNA, pcDNA3. HEK293 cells were employed for the experiment shown in Fig. 3 and Fig. 6, because of its higher transfection efficiency (>80%) compared to that of HepG2 cells (<10%) (data not shown).

### Reagents

Polyinosine-polycytidylic acid (poly I∶C) was purchased from Amersham. Sendai virus (SenV) was kindly provided by Prof. Moon-Jung Song (Korea University).

### Plasmids

All DNA constructs were generated by overlap extension PCR protocols as previously described [43]. HBV Pol expression construct encoding HBV Pol with three copies of Flag tag at its N-terminus has been previously described [42]. The HBV replicon construct (i.e., WT) and its X-null version has been described previously [14]. The HBV C-null and P-null replicons have been described [42]. YMHD mutant of HBV Pol was made by substitution of the aspartic acid residue (i.e., 540D), a constituent of YMDD motif critical for catalysis, to histidine (H), as previously described [44]. Three constructs that was used to express the viral envelope glycoproteins– L-HBsAg, and M-HBsAg, and S-HBsAg, was made by inserting the respective ORFs into pcDNA3 (Invitrogen). The sources of the remaining plasmids are as follows: IRF3-GAL4, IRF7-GAL4, pFR luciferase reporter, IFNβ-Luc reporter (Bowie, Trinity College Dublin, Ireland), TBK1-Flag, IKKε-Flag (Dr. Fitzgerald, University of Massachusetts Medical School, Worcester, MA), Flag-RIG-I (Dr. Fujita, Kyoto University), Flag-TRIF (Akira, Osaka University), HA-TLR3 (InvivoGen), pISRE-Luc reporter (Stratagene), HA-DDX3, AS-DDX3 (K. T. Jeang, N.I.H.), and IRF3-GFP (Garcia-Sastre, Mt. Sinai School of Medicine).

### Western blot analysis

Western blot analysis was performed as described [42]. For the detection of the Flag tagged Pol protein, mouse anti-FLAG M2 antibody (Sigma, 1∶5000) was used. Anti-IRF antibody (Invitrogen) and phospho-specific IRF3 (Ser396) antibody (Cell Signaling) were used to detect IRF3 and phosphorylated IRF3, respectively.

### Reporter gene assays

Promoter induction and transcription factor activation were measured using HepG2 cells seeded onto 24-well or 6-well plates and transfected after 24 h with expression vectors and luciferase reporter gene. For the IRF3/7 assay, an IRF3/7-GAL4 fusion vector was used in combination with the pFR luciferase reporter, as previously described [45].

### Southern blot analysis

Southern blot analysis was performed as previously described [46]. Briefly, the extracted viral DNA was separated by electrophoresis through a 1.3% agarose gel in a 0.5× Tris-acetate-EDTA buffer and then transferred onto a nylon membrane. The nylon membrane was prehybridized and hybridized with a ^32^P-labeled full-length HBV DNA probe in a hybridization solution for 16 h at 65°C. Images were obtained using the phosphoimager (BAS-2500; Fujifilm).

### Co-immunoprecipitation

Immunoprecipitation was performed as described with modifications [47]. Briefly, after transient transfection, the medium was removed and the cells were rinsed twice in cold PBS, incubated for 30 min at 4°C in lysis buffer [50 mM Tris-HCl (pH 7.4), 150 mM NaCl, 1 mM EDTA, 1 mM DTT, 0.2 mM PMSF, and 1% NP-40], and collected by scraping. Cell debris was removed through centrifugation at 10,000×*g* for 10 min at 4°C. Extracts were pre-cleared with protein G–agarose beads for 1 h at 4°C. The primary antibody was added for 1 h at 4°C, and immunoglobulin complexes were collected on protein G–agarose beads for 1 h at 4°C. The beads were washed five times with 1 ml of lysis buffer. Protein complexes were recovered by boiling in Laemmli sample buffer and analyzed by SDS–PAGE.

### Confocal imaging

HEK293 cells were grown on 18-mm coverslips in 12-well plates and transfected with 2.5 µg of total DNA. At 48 h after transfection, cells were fixed with 3.7% formaldehyde. Slides were mounted onto glass slides with ProLong Antifade Kit (Molecular Probes) and examined by confocal microscopy (LSM 510 Meta; Carl Zeiss, Germany).

### Accession numbers

HBV ayw subtype: V01460 J02203

DDX3: accession NM_001356.3

## Supporting Information

Figure S1 The Map of HBV replicon constructs used in this study. WT represents the 1.2mer over-the-genome length HBV replicon construct. Three ORFs are drawn on the pregenomic RNA with two stem-loop structures (epsilon or ε), but S ORF is omitted for clarity. Three mutant replicon constructs including the P-null, C-null, and X-null constructs are drawn with the introduced stop codons denoted by dots. The ORF with dashed line denotes the inactivated ORF.
(0.10 MB TIF)

Figure S2 Southern blot analysis of viral DNA isolated from cytoplasmic capsids. Cells were transfected either with the wild-type HBV replicon or the P-null replicon along with an increasing amount of the Pol expression construct: 1.0, 2.0, and 4.0 µg per 6-well plate, which are equivalent to 0.5, 1.0, and 2.0 µg per 12-well plate, respectively. Viral DNAs isolated from cytoplasmic capsids were analyzed by Southern blot analysis. The viral replication DNA intermediates RC (relaxed circular) and DL (duplex linear) DNA are denoted. In parallel, HBV Pol was examined by Western blot analysis with anti-Flag antibody.
(0.37 MB TIF)

Figure S3 DDX3 is essential for TBK1/IKKε-dependent IRF activation in HepG2 cells. To knock down endogenous DDX3, an antisense DDX3 construct was used HepG2 cells were transfected with either IKKε (A) or TBK constructs (B) and the IRF3 reporter construct, along with increasing doses of the antisense DDX3 construct (i.e. AS-DDX3). IRF3 activation was monitored as shown in Fig. 4. IRF3 signaling was blocked by AS-DDX3 transfection indicating that DDX3 is essential for IRF3 signaling in HepG2 cells. It was noted that the impact of DDX3 on TBK-mediated IRF3 activation was less than that seen on IKKε-mediated IRF3 activation.
(0.07 MB TIF)
