# Supplementary figures and images for: Hepatitis B Virus Polymerase Blocks Pattern Recognition Receptor Signaling via Interaction with DDX3: Implications for Immune Evasion
Source: PLoS Pathog. 2010 Jul 15;6(7):e1000986. doi: 10.1371/journal.ppat.1000986 (PMC2904777; doi:10.1371/journal.ppat.1000986)

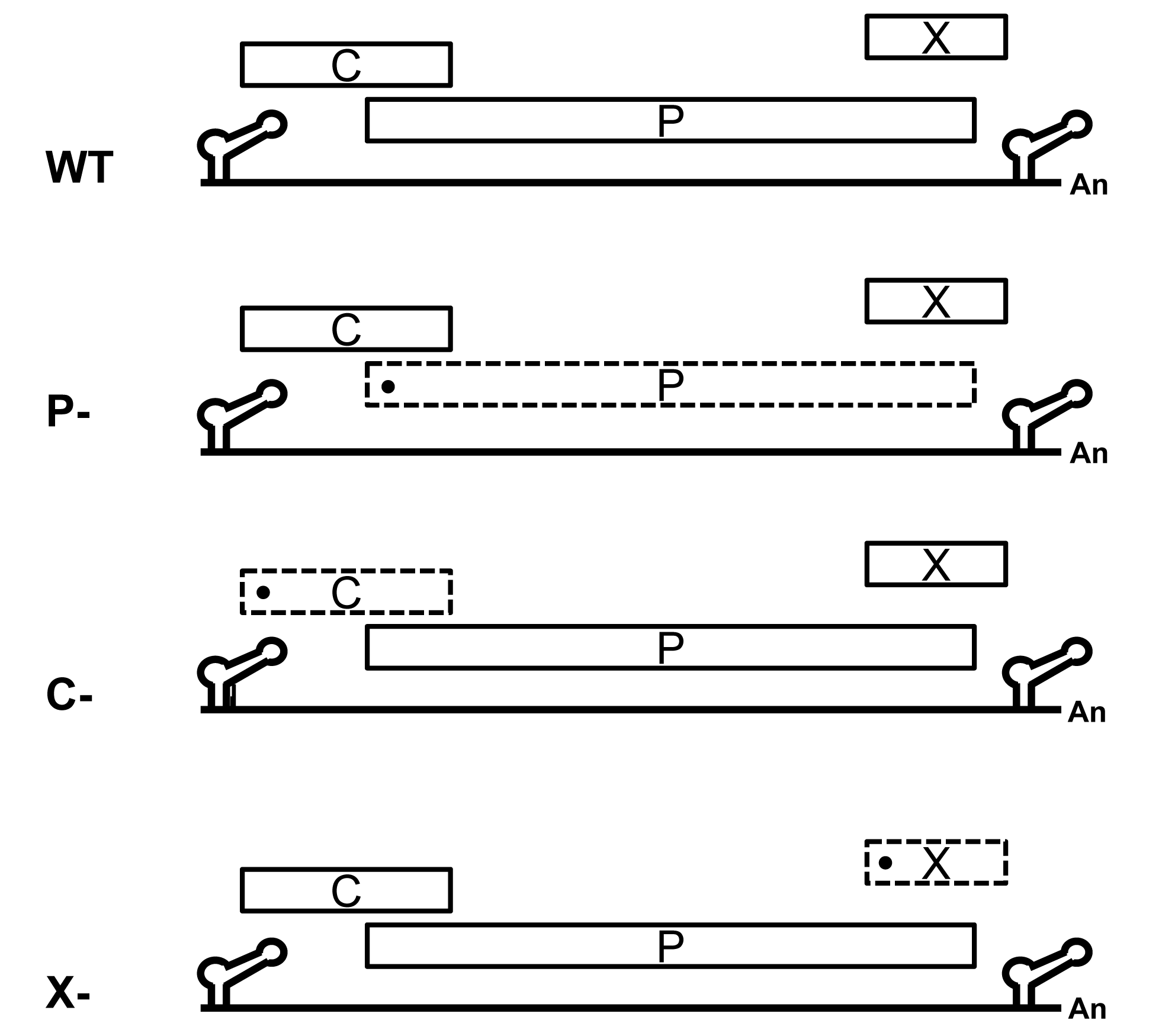

Supplement: Figure S1 — The Map of HBV replicon constructs used in this study. WT represents the 1.2mer over-the-genome length HBV replicon construct. Three ORFs are drawn on the pregenomic RNA with two stem-loop structures (epsilon or ε), but S ORF is omitted for clarity. Three mutant replicon constructs including the P-null, C-null, and X-null constructs are drawn with the introduced stop codons denoted by dots. The ORF with dashed line denotes the inactivated ORF. (0.10 MB TIF) [file ppat.1000986.s001.tif]

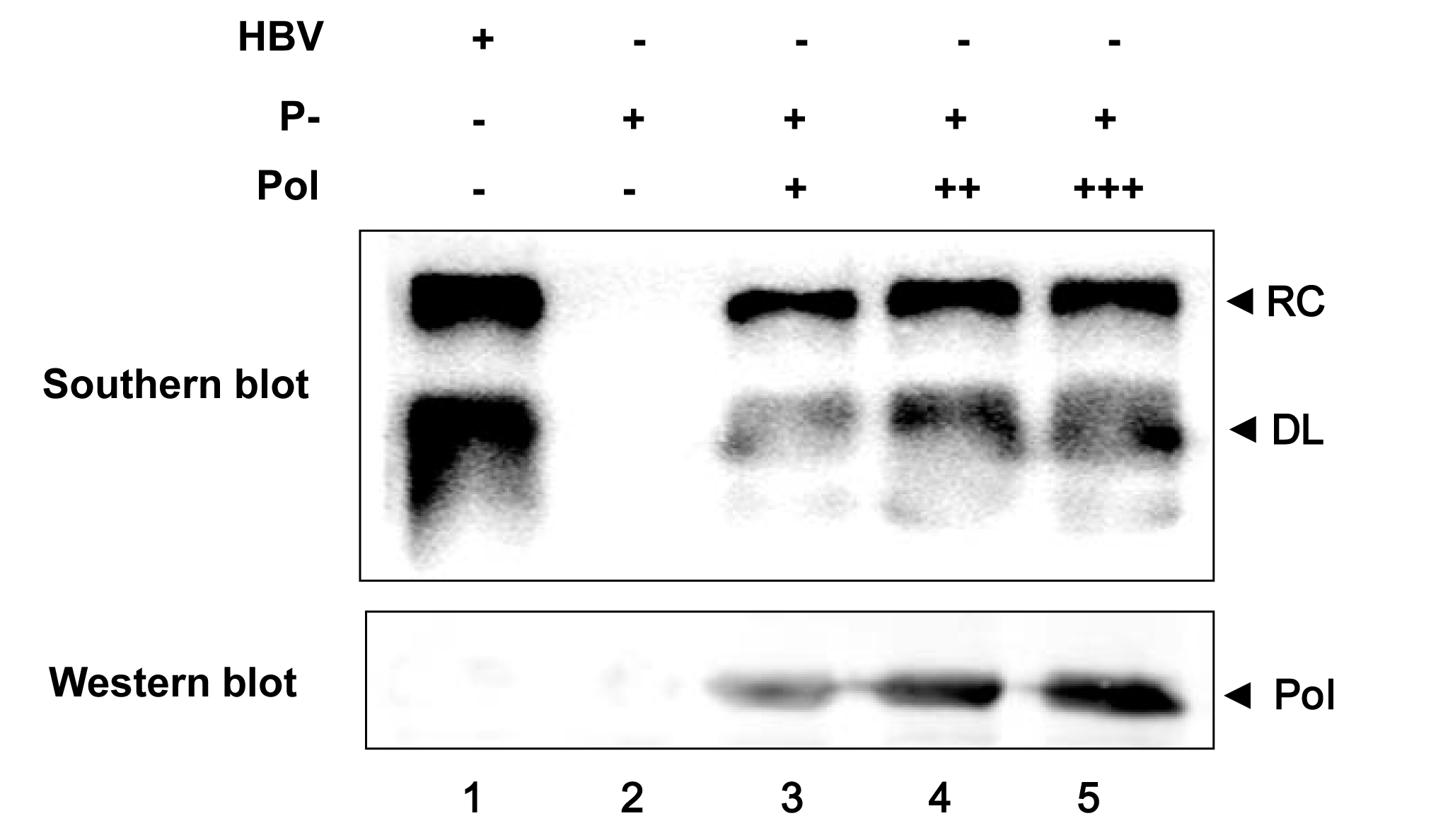

Supplement: Figure S2 — Southern blot analysis of viral DNA isolated from cytoplasmic capsids. Cells were transfected either with the wild-type HBV replicon or the P-null replicon along with an increasing amount of the Pol expression construct: 1.0, 2.0, and 4.0 µg per 6-well plate, which are equivalent to 0.5, 1.0, and 2.0 µg per 12-well plate, respectively. Viral DNAs isolated from cytoplasmic capsids were analyzed by Southern blot analysis. The viral replication DNA intermediates RC (relaxed circular) and DL (duplex linear) DNA are denoted. In parallel, HBV Pol was examined by Western blot analysis with anti-Flag antibody. (0.37 MB TIF) [file ppat.1000986.s002.tif]

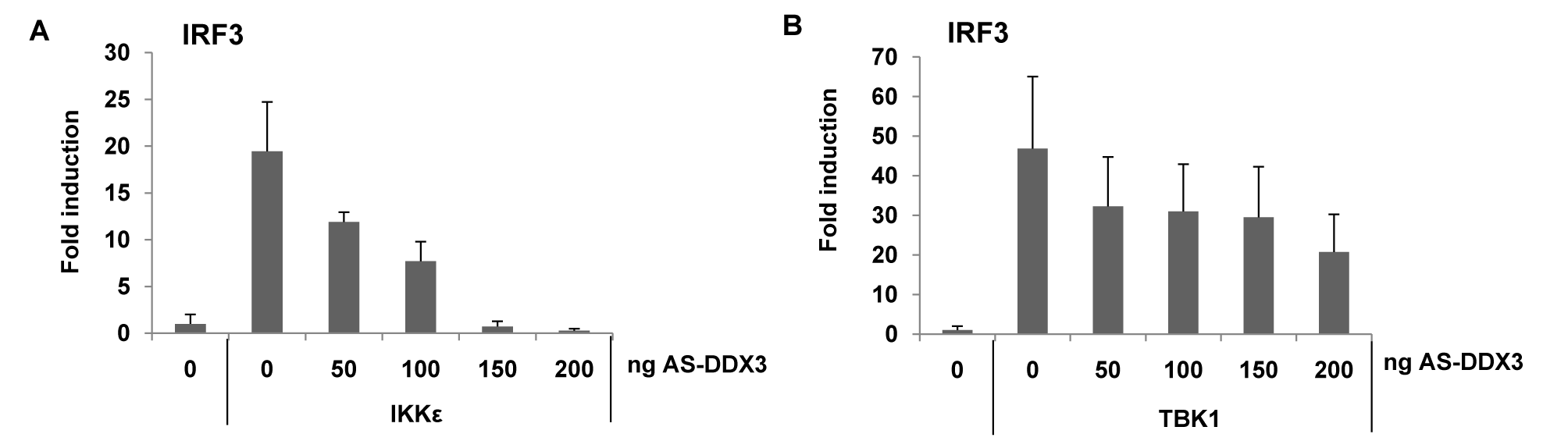

Supplement: Figure S3 — DDX3 is essential for TBK1/IKKε-dependent IRF activation in HepG2 cells. To knock down endogenous DDX3, an antisense DDX3 construct was used HepG2 cells were transfected with either IKKε (A) or TBK constructs (B) and the IRF3 reporter construct, along with increasing doses of the antisense DDX3 construct (i.e. AS-DDX3). IRF3 activation was monitored as shown in Fig. 4. IRF3 signaling was blocked by AS-DDX3 transfection indicating that DDX3 is essential for IRF3 signaling in HepG2 cells. It was noted that the impact of DDX3 on TBK-mediated IRF3 activation was less than that seen on IKKε-mediated IRF3 activation. (0.07 MB TIF) [file ppat.1000986.s003.tif]
